# Gut microbiota of white-headed black langurs (*Trachypithecus leucocephalus*) in responses to habitat fragmentation

**DOI:** 10.3389/fmicb.2023.1126257

**Published:** 2023-02-13

**Authors:** Ying Lai, Yanqiong Chen, Jingjin Zheng, Zheng Liu, Dengpan Nong, Jipeng Liang, Youbang Li, Zhonghao Huang

**Affiliations:** ^1^Key Laboratory of Ecology of Rare and Endangered Species and Environmental Protection (Guangxi Normal University), Ministry of Education, Guilin, China; ^2^Guangxi Key Laboratory of Rare and Endangered Animal Ecology, Guangxi Normal University, Guilin, China; ^3^College of Life Sciences, Guangxi Normal University, Guilin, China; ^4^Administration Center of Guangxi Chongzuo White-headed Langur National Nature Reserve, Chongzuo, China

**Keywords:** white-headed black langur, gut microbiota, habitat fragmentation, intersite variations, community assembly, limestone forest

## Abstract

The white-headed black langur (*Trachypithecus leucocephalus*) is exclusively distributed in the karst forests and is critically endangered owing to habitat fragmentation. Gut microbiota can provide physiological data for a comprehensive study of the langur’s response to human disturbance in the limestone forest; to date, data on spatial variations in the langurs’ gut microbiota are limited. In this study, we examined intersite variations in the gut microbiota of white-headed black langurs in the Guangxi Chongzuo White-headed Langur National Nature Reserve, China. Our results showed that langurs in the Bapen area with a better habitat had higher gut microbiota diversity. In the Bapen group, the Bacteroidetes (13.65% ± 9.73% vs. 4.75% ± 4.70%) and its representative family, Prevotellaceae, were significantly enriched. In the Banli group, higher relative abundance of Firmicutes (86.30% ± 8.60% vs. 78.85% ± 10.35%) than the Bapen group was observed. Oscillospiraceae (16.93% ± 5.39% vs. 16.13% ± 3.16%), Christensenellaceae (15.80% ± 4.59% vs. 11.61% ± 3.60%), and norank_o__Clostridia_UCG-014 (17.43% ± 6.64% vs. 9.78% ± 3.83%) were increased in comparison with the Bapen group. These intersite variations in microbiota diversity and composition could be accounted for by differences in food resources caused by fragmentation. Furthermore, compared with the Banli group, the community assembly of gut microbiota in the Bapen group was influenced by more deterministic factors and had a higher migration rate, but the difference between the two groups was not significant. This might be attributed to the serious fragmentation of the habitats for both groups. Our findings highlight the importance of gut microbiota response for the integrity of wildlife habitats and the need in using physiological indicators to study the mechanisms by which wildlife responds to human disturbances or ecological variations.

## 1. Introduction

Animals commonly depend on physiological regulation in case of being unable to make further adaptations by shifting behavior when facing harsh existential conditions (Wong and Candolin, 2015; Bahram et al., 2016). Of the various physiological expressions, gut microbiota can be considered an effective indicator of the physiology and even the health status of wild animals (McManus et al., 2021). The dynamic balance established between the gut microbiota and host in their long-term interactions is related to energy acquisition and nutritional metabolism (Wu et al., 2011), which affects the immune system (Matson et al., 2021), nervous system (Cryan et al., 2019), and growth (Groer et al., 2014). Intense influences may severely disrupt the gut microbiota, which normally remains relatively stable, causing it to lose basic resistance and resilience and eventually leading to various diseases (Downing and Leibold, 2010; de Mazancourt et al., 2013; Fackelmann et al., 2021).

The gut microbiota is heavily dependent on the genetics of the host; however, environmental factors also shape the structures and functions of the gut microbiota, even outweighing the influence of the host’s anatomical and physiological characteristics (Dominguez-Bello et al., 2010; Rothschild et al., 2018). Conspecific animals distributed in habitats of different quality have unique gut microbiota (Amato et al., 2013; Barelli et al., 2015). For example, black howler monkeys (*Alouatta pigra*; Amato et al., 2013) and Udzungwa red colobus monkeys (*Procolobus gordonorum*; Barelli et al., 2015) exhibit a lower abundance and diversity of gut microbiota when inhabiting areas with heavier fragmentation compared with those inhabiting habitat with less fragmentation. There are significant differences in gut microbiota of rhesus macaques (*Macaca mulatta*) distributed in different geographical populations being grouped by altitude conditions, which is manifested by the production of new and unique microbiota (Zhao et al., 2018). Additionally, a decreased diversity in the gut microbiota of primates has been observed in gray–brown mouse lemurs (*Microcebus griseorufus*), which may be associated with varying degrees of human invasion of each habitat, occurring in Bale monkeys (*Chlorocebus djamdjamesis*) as well (Trosvik et al., 2018; Wasimuddin et al., 2022). The abundance of beneficial gut microbiota and functional metabolism genes is linked to the health of the species, with those in worse habitats showing a decreasing trend, which indicates that these individuals have a more potential disease risk (Amato et al., 2013; Barelli et al., 2015; Trosvik et al., 2018; Wasimuddin et al., 2022). Furthermore, the differences in the gut microbiota of animals living in different habitats are an outcome of adaptive alterations in response to ecological changes and behavioral adjustments, notably dietary composition (Wu et al., 2011; McManus et al., 2021).

By supplying a matrix for a given microbiota, diet plays a key role in shaping the host gut microbiota, including assisting hosts in selecting and expanding their corresponding degradation ability to alter the diversity and composition of the gut microbiota (Wu et al., 2011). For instance, herbivores exhibit a higher gut diversity than carnivores and omnivores owing to their more complex diets (Ley et al., 2008; Szekely et al., 2010; Deehan et al., 2020). Specifically, the Bacteroidetes has genes encoding enzymes that hydrolyze complicated plant polysaccharides (Grondin et al., 2017), and the Firmicutes is considered to have lignin-degrading functions (Liu et al., 2019; Que et al., 2022). The hosts meet their major energy and nutritional demands with the help of gut microbiota that convert foods into short-chain fatty acids (SCFAs; Turnbaugh et al., 2006; Sun et al., 2022). Hence, both phyla are often represented in large proportions in the gut microbiota of herbivores (De Filippis et al., 2016). In the wild primates, a higher relative abundance of Bacteroidetes has been linked to the digestion of high-quality foods, such as fruits, young leaves, and buds, which are distributed more widely in high-quality habitats (Li et al., 2021; Xia et al., 2021). In contrast, a higher abundance of Firmicutes is regarded as a response to low-quality habitats that contain fewer high-quality food resources (Li et al., 2021; Xia et al., 2021), as observed in other typical folivorous primates that must degrade crude cellulose and lignin, such as François’s langur (*Trachypithecus francoisi*; Chen et al., 2020) and the silvered langur (*Trachypithecus cristatus*; Le et al., 2019; Que et al., 2022). Therefore, data on relationship between the host’s gut microbiota, diet and habitat could provide insights into adaptation strategy, consequently facilitating effective wildlife conservation.

Whether the host is sensitive to the environment touches on the process of microbial community assembly (Zhou and Ning, 2017). The niche and neutral theories are the two theoretical frameworks for understanding microbial community assembly (Dini-Andreote et al., 2015). The neutral theory states that stochastic processes are correlated with the birth, death, migration, and ecological drift of microbiota community, whereas the niche theory argues that deterministic processes are linked to abiotic and biotic factors (Fargione et al., 2003; Zhou et al., 2013). Whether deterministic or stochastic processes are dominant in community assembly has been controversial (Chen et al., 2019; Li et al., 2019). The relative importance of stochastic and deterministic processes can be quantified using the neutral community model (NCM; Sloan et al., 2006). For example, the NCM reveals that deterministic and stochastic processes jointly shape microeukaryotic community assembly in rivers under human disturbance (Gad et al., 2020). However, this assembly in subtropical rivers is shaped by stochastic processes because the water is more complex and changeable (Chen et al., 2019). In contrast, a study on rhesus macaques with different foods in altered seasons confirmed the dominant role of deterministic processes in microbiota assembly owing to environmental filtering (Liu et al., 2022). The variations in the importance of deterministic and stochastic processes in the microbiota are likely habitat-dependent; therefore, investigating the assembly process in biological communities is important to explain the formation and maintenance mechanisms of biodiversity in the microbiota (Zhou and Ning, 2017; Heys et al., 2020).

White-headed black langurs (*Trachypithecus leucocephalus*) are exclusively distributed in the limestone forest in Southwest Guangxi, China, and are listed as critically endangered on the IUCN Red List (Bleisch and Long, 2020). These langurs are leaf-eating animals that prefer young leaves (Huang et al., 2008b). These animals face severe habitat fragmentation aggravated by human disturbance, which is threatening their survival (Huang, 2002; Li and Rogers, 2005). The Guangxi Chongzuo White-headed Langur National Nature Reserve comprises four areas, namely, Dalin, Tuozhu, Banli, and Bapen. This study was conducted in Banli and Bapen. The Banli area is more severely fragmented and has lower plant diversity than the Bapen area because of increased human disturbance (Huang et al., 2008a; Huang et al., 2017). Previous studies have focused on the behavioral ecology of white-headed black langurs (Zhou et al., 2011; Huang et al., 2017; Zhang et al., 2021) and have provided data on their adaptability to habitat fragmentation. However, the mechanism of the langurs’ physiological response to habitat fragmentation remains unclear. Hence, in this study, we analyzed the gut microbiota from 203 fecal samples of white-headed black langurs. We first described the structural features of the gut microbiota and then further compared the intersite variations in the diversity and composition of the gut microbiota. Finally, we assessed the relative importance of the community assembly process of the gut microbiota by applying the NCM. We tested the following predictions:

1. A positive correlation has been found between diet diversity and gut microbiota (Heiman and Greenway, 2016; Frankel et al., 2019). More-fragmented habitats with lower vegetation diversity stimulate Banli group to forage and increase the diversity of their diets (Huang et al., 2017; Zhang et al., 2021). We, thus, predicted that the diversity of the gut microbiota in the Banli group would be higher than that in the Bapen group.
2. Young leaves in fragmented habitats are commonly limited (Zhou et al., 2011). Banli group suffering deeper habitat fragmentation will rely more heavily on low-quality foods, such as mature leaves (Li et al., 2016). We, thus, predicted that langurs living in Banli would have a higher relative abundance of cellulose-degrading bacteria.
3. Lower vegetation diversity may intensify the competition for resources (Li et al., 2022). Banli has lower vegetation diversity (Huang et al., 2008a). We, thus, predicted that the community assembly of the langurs’ gut microbiota in Banli area would be more affected by deterministic processes.

## 2. Materials and methods

### 2.1. Study site, study subjects, and sample collection

This study was performed in the Guangxi Chongzuo White-headed Langur National Nature Reserve (107°16′53″–107°59′46″E, 22°10′43″–22°36′55″N), which is covered by limestone forest with an altitude ranging from 400 to 600 m (Guangxi Forestry Department, 1993). Fecal samples for this study were collected from nine groups in Banli and Bapen. We collected 155 fecal samples from Banli between December 2020 and January 2021 and June and July 2021. We collected 48 samples from Bapen during July and November 2021 (Supplementary Table 1). White-headed black langurs use caves and/or crevices in cliffs as their permanent sleeping sites and defecate at the cave edges before leaving their sleeping sites in the morning. We collected feces under the cliffs after the langurs had left. While collecting the samples, we wore sterile gloves and used sterilized bamboo sticks to obtain ~3–5 g of the internal part that was uncontaminated and placed it in sterile collection tubes. After making clear marks, we immediately placed the samples in a dry ice box and then transferred them to a −80°C ultra-low–temperature refrigerator for storage until further DNA extraction.

### 2.2. Ethics approval

We collected langur fecal samples with the permission of the Administration Center of Guangxi Chongzuo White-headed Langur National Nature Reserve. This study did not involve any invasive animal tissue procedures. Furthermore, we collected the samples after the langurs left their sleeping sites to avoid any stress reactions caused by the collection.

### 2.3. DNA extraction, PCR amplification, and sequencing

Total DNA was extracted from fecal samples of white-headed black langurs using an E.Z.N.A.^®^ Soil DNA Kit (Omega Bio-Tek, America). A NanoDrop 2000 (Thermo Fisher Scientific, America) was used to detect the concentration and purity of the DNA, and 1% agarose gel electrophoresis was used to detect the extraction quality of the DNA. The TransGen AP221-02 reaction system (TransGen, China) comprised 4 μL of 5× FastPfu buffer, 4 μL of 2.5 mM dNTPs, 0.8 μL of forward primer (5 μm) and reverse primer (5 μm), 0.4 μL of FastPfu polymerase, 0.2 μL of BSA, 10 ng of template DNA, and enough ddH_2_O to bring the total amount of reagent to 20 μL. In this system, highly specific amplification primers were used (338F:5′-ACTCCTACGGGAGGCAGCAG-3′ and 806R:5′-GGACTACHVGGGTWTCTAAT-3′; Mori et al., 2014). The amplified DNA was first denatured at 95°C for 3 min, then subjected to 28 PCR cycles (denaturation at 95°C for 30 s, annealing at 53°C for 30 s, and extension at 72°C for 45 s), and finally extended at 72°C for 10 min to obtain amplification products located in the 16S rDNA V3–V4 region using a PCR amplifier model ABI GeneAmp^®^ 9700 (ABI, America). Three replicates of each sample were run, and products from the same sample were mixed and detected using 2% agarose gel electrophoresis and recovered using an AxyPrep DNA Gel Extraction Kit (Axygen, America). The PCR products were detected and quantified using a Quanti Fluor™-ST Blue Fluorescence Quantification System (Promega, America), after which the corresponding proportion was mixed as per the sequencing volume requirement of each sample. According to the fluorescence quantitative measurement results, a NEXTFLEX Rapid DNA-Seq kit (Bio Scientific, America) was used to construct a sequencing library. The sequencing platform used was an Illumina Miseq PE 300 (Illumina, America).

### 2.4. Bioinformatics and statistical analysis

The following procedures were performed on Majorbio Cloud Platform^1^. The raw sequences were quality-controlled using Trimmomatic. This step included removing repetitive, low-quality (<20 bp) sequences containing primer linkers and sequences with a high proportion of N and then filtering out reads of <50 bp after quality control. The PE reads obtained from sequencing were spliced with Flash 1.2.11^2^ (Magoč and Salzberg, 2011) according to the overlap relationship. The redundant, low-abundance sequences were removed with Usearch 11^3^ (Edgar, 2010), and the chimeras were removed with UCHIME (Edgar et al., 2011) to obtain valid sequences. The valid sequences of all samples were clustered using Uparse 11^4^ (Edgar, 2013) at a threshold of 97% to obtain the OTUs. The OTU representative sequences were generated using Qiime 1.9.1^5^ (Caporaso et al., 2010), and valid sequences with >97% similarity to the representative sequences were selected to generate the original OTU tables. The obtained OTUs were compared with the 16S rRNA SILVA 138 bacterial database^6^ for species classification annotation using RDP Classifier,^7^ and the classification confidence level was set to 80%. The samples were leveled twice using the minimum number of sequences, and only OTUs of bacterial domains were retained, finally yielding the OTU tables for subsequent analysis.

Alpha diversity analysis was performed using Mothur 1.30.3.^8^ The dilution curves were used to determine whether the data for this sequencing were sufficient and reasonable. Values of *inv*Simpson represent the reciprocal of Simpson index, and the variations in values with the Shannon index are proportional to species diversity. The ACE and Chao indexes indicate the species richness of the community. The principal co-ordinate analysis (PCoA) reflects the similarity or variability of the community composition in the samples. The distance between different sample points was calculated using Qiime 1.9.1. Significant differences were analyzed for sample species between two sites using a Wilcoxon rank–sum test with a confidence interval of 95%, and the results were expressed as corrected *p*-values. Based on the Kyoto Encyclopedia of Genes and Genomes (KEGG) database, the functional prediction of gut microbiota in fecal samples was performed by PICRUSt 2 (version 2.2.0^9^; Douglas et al., 2020). According to the information obtained from the three levels of the KEGG pathway, the abundance of each level was calculated to obtain the abundance table of KEGG pathway. Intersite differences in functional pathways were analyzed using the Mann–Whitney *U*-test.

The following operations were performed with R 4.1.2: The alpha diversity indexes were converted into the form of log_10_ (x) (Warton and Hui, 2010). They were visualized as box plots and used for generalized linear mixed model (GLMM) calculations. The GLMM was used to compare intersite differences in the alpha diversity of the gut microbiota of white-headed black langurs. In this model, the geography was set as a fixed factor, the indices as response variables, and the different monkey groups as random factors. The differences between the two models with and without fixed effects were compared using ANOVA to determine the effect of fixed factors on the response variables.

We then constructed the NCM based on an OTU table to demonstrate the relative importance of stochastic processes on gut microbiota community assembly (Sloan et al., 2006). This model estimated the migration rate of the gut microbial community (indicated by the *m* value) and the impact of random effects on the construction process (indicated by *R*^2^). The value of *m* is inversely proportional to the dispersal limitation, which shows that abundant taxa are generally higher than rare taxa because the latter are more likely to disappear from a single host owing to ecological drift. Within the range of 0–1, *R*^2^ represents the stochastic effect and the remaining part represents the deterministic effect (1 − *R*^2^).

Using the randomForest package, massive decision trees were constructed after processing the data according to the random and put-back sampling principle (Breiman, 2001). Objects were classified sequentially to obtain the random forest model. The relative importance of bacterial taxa was expressed using Mean Decrease Gini and ranked, with larger values being more important. To ensure the performance of the random classifier, we performed the following steps: First, 70% fraction of the sample was designated as the training set and the remaining 30% as the test set, which served as the basis for evaluating the model performance. Next, a 10-fold cross-validation was performed and repeated five times to avoid uncertain evaluation results. Eventually, the area under the curve (AUC) values were obtained using the pROC package (Robin et al., 2011), considering values ranging from 0.5 (nonsense classification) to 1 (perfect classification; Robin et al., 2011).

## 3. Results

### 3.1. Variations in gut microbiota composition: Banli group possessed more unique taxa and richer bacterial taxa of Firmicutes than Bapen group

After the quality control, a total of 11,291,593 optimized sequences were obtained from 203 fecal samples, with an average of 55,624 ± 13,334 sequences per sample (Supplementary Table 2). The OTU table after flattening revealed 3,091 OTUs, 35 phyla, 384 families, and 778 genera. Good’s coverage estimators for all samples ranged from 98.40% to 99.68% (99.38% ± 0.22%; Supplementary Table 3), which indicated that the sequencing results were representative of the actual occurrence of microbial species in the samples. The Shannon (Supplementary Figure 1A) and Sob curves (Supplementary Figure 1B) showed that the curves of all samples were asymptotic, which signified that the amount of sequencing data was sufficient to reflect the majority of microbial diversity information in the samples.

The langurs from the Banli and Bapen groups shared 1878 OTUs, 31 phyla, and 284 families. The Banli group possessed 1,024 unique OTUs and 79 unique families, and the Bapen group exhibited 189 unique OTUs and 21 unique families. Moreover, all groups had two unique phyla (Supplementary Figure 2).

**Figure 1:**
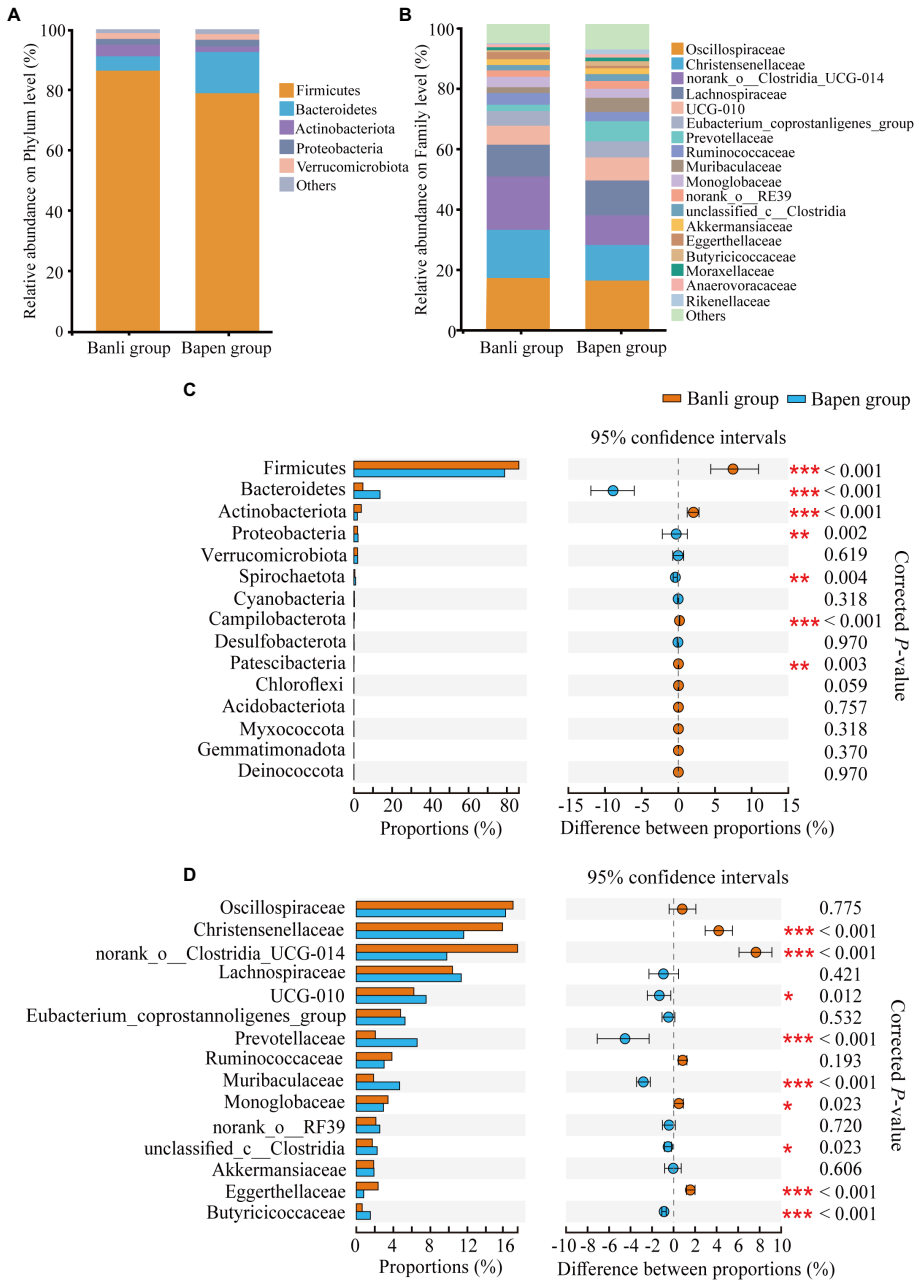
Composition of the gut microbiota at the phylum **(A)** and family **(B)** levels. All taxa with a relative abundance of <1% were classified as “others”. The composition difference analysis in the gut microbiota community at the phylum **(C)** and family **(D)** levels. Only the top 15 bacterial taxa are displayed. Significant difference was expressed by “*” for *p* < 0.05, “**” for *p* < 0.01, and “***” for *p* < 0.001.

At the phylum level, all samples were occupied by Firmicutes (Banli group: 86.30% ± 8.60% vs. Bapen group: 78.85% ± 10.35%), Bacteroidetes (4.75% ± 4.70% vs. 13.65% ± 9.73%), and Actinobacteria (3.88% ± 3.56% vs. 1.82% ± 2.04%), which accounted for >94% of the total relative abundance (Figure 1A). At the family level, the top three included Oscillospiraceae (Banli group: 16.93% ± 5.39% vs. Bapen group: 16.13% ± 3.16%), Christensenellaceae (15.80% ± 4.59% vs. 11.61% ± 3.60%), and norank_o__Clostridia_UCG-014 (17.43% ± 6.64% vs. 9.78% ± 3.83%; Figure 1B). Other taxonomic groups at the phylum and genus levels are shown in Supplementary Tables 4, 5.

Dominant phyla and families in the samples were analyzed for intergroup differences using the Wilcoxon rank–sum test. The results showed that the relative abundance of Firmicutes and Actinobacteria in the Banli group was significantly higher than that in the Bapen group at the phylum level, whereas Bacteroidetes and Proteobacteria in the Bapen group were significantly increased compared with the Banli group (Figure 1C). At the family level, the relative abundances of Christensenellaceae and norank_o__Clostridia_UCG-014 in the microbiota were significantly higher than those of the Bapen group, whereas the relative abundances of UCG-010, Prevotellaceae, and Butyricicoccaceae in the Bapen group were significantly higher than those of the Banli group (Figure 1D). Details of the various species at both taxonomic levels are listed in Supplementary Tables 4, 5.

**Figure 2:**
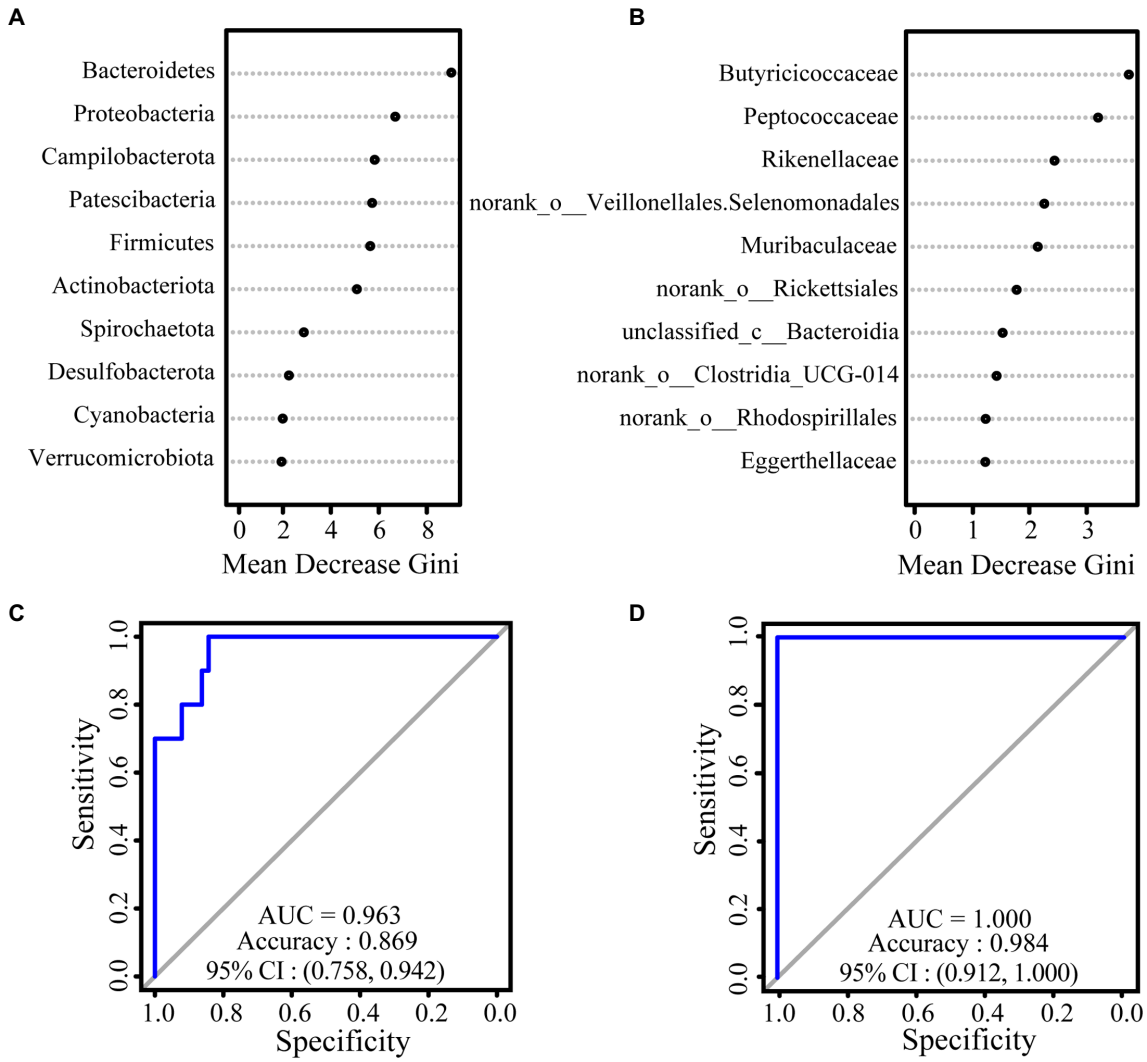
The results of relative importance ranking based on values of Mean Decrease Gini at the phylum **(A)** and family **(B)** levels. ROC curve used to test the effect of the classifier of random forest model at the phylum **(C)** and family **(D)** levels. The closer the difference between the value of AUC and 1, the better the result of classification.

We used a random forest model to construct classifiers to rank the importance of gut microbiota, which promotes the formation of intersite variations. At the phylum level, the most important taxa contributing to intersite variations in the gut microbiota of white-headed black langurs were Bacteroidetes, followed by Proteobacteria and Campylobacterota. At the family level, these variations were accounted for by Butyricoccaceae, Peptococcaceae, and Rikenellaceae. The AUC values of the phylum and family levels were 0.963 and 1.000, respectively, which indicate an exceptional classification (Figure 2).

### 3.2. Variations in gut microbiota diversity: Bapen group had higher alpha diversity index than Banli group

**Figure 3:**
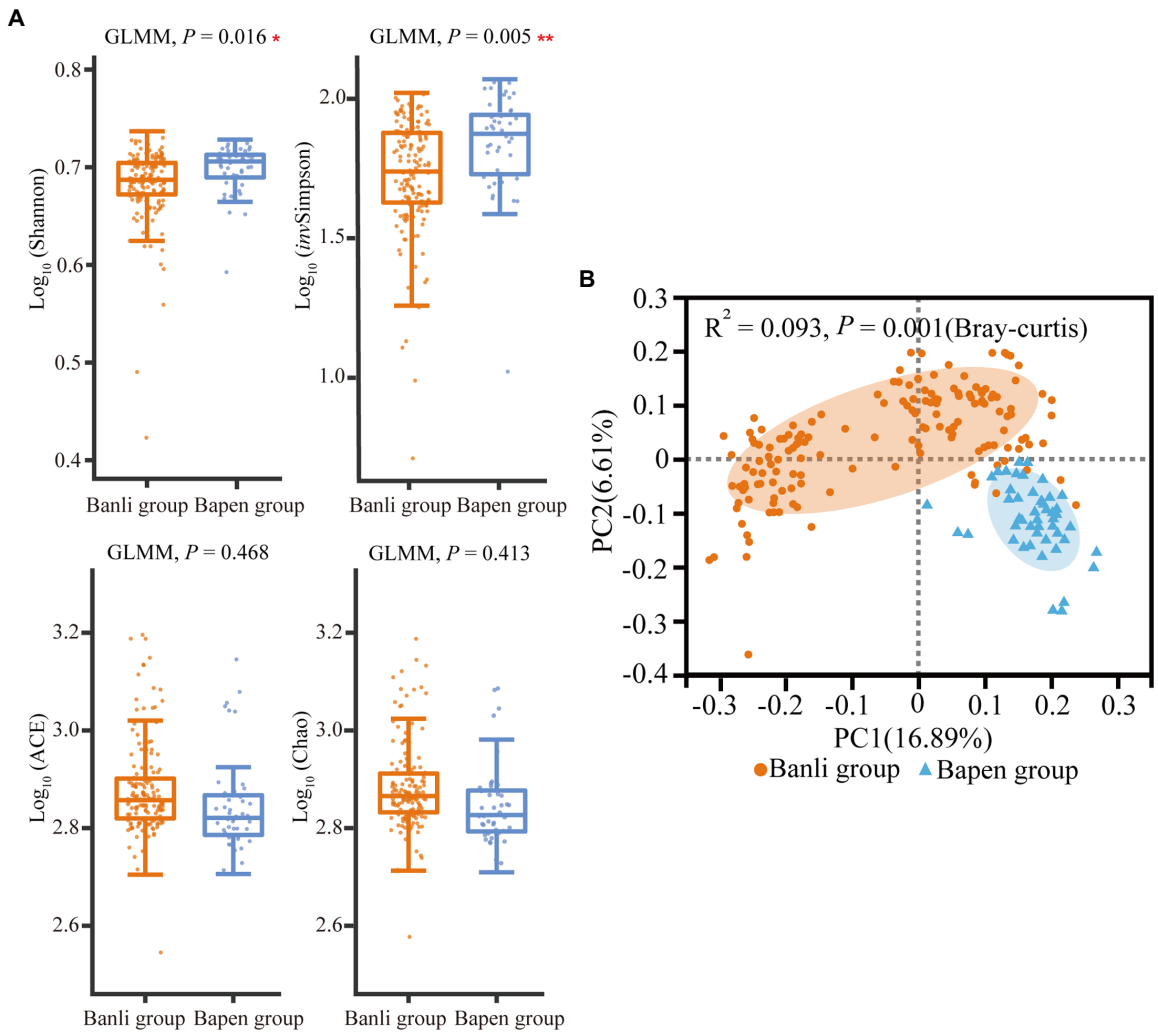
Compared with the alpha diversity in gut microbiota between the Banli and Bapen groups **(A)**. Significant difference was expressed by “*” for *p* < 0.05, “**” for *p* < 0.01, and “***” for *p* < 0.001. Regional comparison of gut microbiota beta diversity based on OTUs (Tested by Adonis) **(B)**.

According to the results of alpha diversity analysis, the Shannon index (Banli group: 4.821 ± 0.370 vs. the Bapen group: 4.984 ± 0.264) and *inv*Simpson index (57.575 ± 22.396 vs. 73.890 ± 23.367) of the gut microbiota of langurs in the Banli and Bapen group differed significantly (Shannon: *χ*^2^ = 5.792, df = 1, *p* = 0.016; *inv*Simpson: *χ*^2^ = 7.734, df = 1, *p* = 0.005). However, the ACE index and Chao index did not show significant intersite variations (Figure 3A). The detailed results of mean and SD values and GLMM are shown in Supplementary Tables 3, 6, respectively.

Results of the PCoA based on Bray–Curtis showed that the structures of the gut microbiota of langurs differed significantly (*R*^2^ = 0.093, *p* = 0.001) between geographic groups (Figure 3B).

### 3.3. Variations in the functional profiles of gut microbiota: Banli group had richer functional pathways than Bapen group

**Figure 4:**
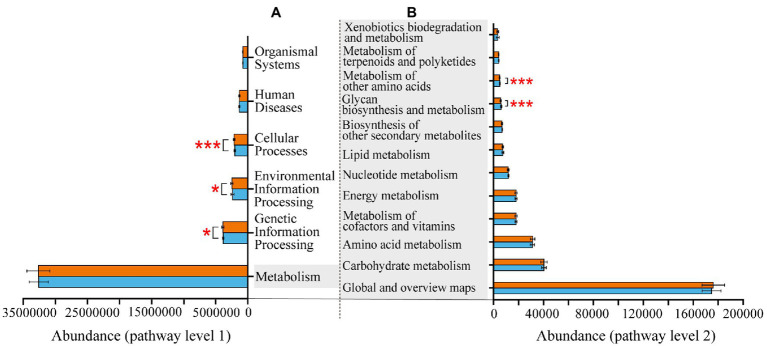
Differences in the functional profiles prediction in gut microbiota of white-headed black langurs in pathway level 1 **(A)** and pathway level 2 **(B)**. Significant difference was expressed by “*” for *p* < 0.05, “**” for *p* < 0.01, and “***” for *p* < 0.001. The orange bar represents Banli group, and the blue one represents Bapen group.

At KEGG pathway level 1, there were three pathways, namely, cellular processes, environmental information processing and genetic information processing, more enriched in Banli group. The remaining three pathways had no significant difference between the two groups. At KEGG pathway 2, the pathways related to metabolism were more abundant in the Bapen group than in the Banli group. Specifically, metabolism of other amino acids and glycan biosynthesis and metabolism were more enriched in the Bapen group than in the Banli group (Figure 4).

### 3.4. Community assembly of gut microbiota: Two groups shared similar explanation and migration rate

**Figure 5:**
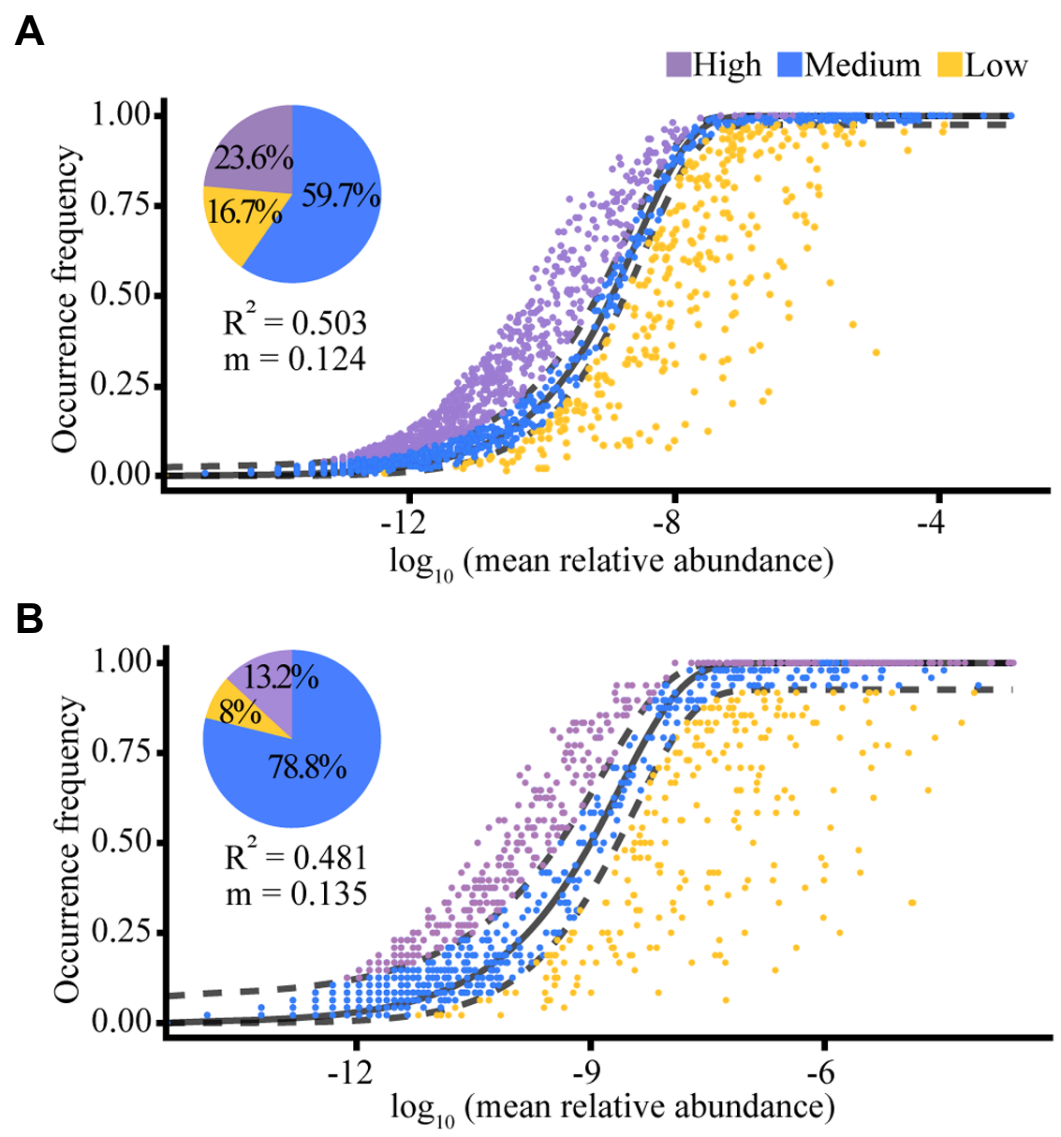
The quantitative results of stochastic processes in the community assembly of the gut microbiota in Banli group **(A)** and Bapen group **(B)**. Random effects on the community construction of gut microbiota were expressed by “*R*^2^”. The migration rate of species for the whole microbiota community is denoted by “*m*”. The solid black line indicates the best fitness between the actually occurring frequency of OTUs and the theoretical predicted frequency of the model. Field between the two black dashed lines means the 95% confidence interval, beyond which is an outlier.

According to the results of the NCM, random effects explained 50.3% (for the Banli group) and 48.1% (for the Bapen group) of the community assembly of the white-headed black langurs’ gut microbiota. The migration rate of gut microbiota was higher in the Bapen group (*m* = 0.135) than in the Banli group (*m* = 0.124). Of the gut microbiota of the Banli group, 59.7% were within the theoretical prediction range of the random effects calculated by the model, whereas 23.6% were above this value and 16.7% were below it. The proportion of gut microbiota in the Bapen group that fell within the interval of theoretical predictions as measured by the model was 78.8%, with 13.2% above this limit and 8.0% below it (Figure 5).

## 4. Discussion

### 4.1. Characteristics of the gut microbiota

The predominant taxa in the gut microbiota of white-headed black langurs in the fragmented habitats are Firmicutes and Bacteroidetes. Previous studies found similar results in colobine monkeys, such as Sichuan snub-nosed monkeys (*Rhinopithecus roxellana*; Su et al., 2016) and Udzungwa red colobus monkeys (Barelli et al., 2015). The sympatric rhesus macaques and François’ langurs also follow a similar pattern (Chen et al., 2020). This result might be related to the fragmentation of its habitat in different degrees. Generally, degradation of habitat quality leads to a scarcity of high-quality food resources, such as fruits (Zhao et al., 2018). To satisfy the basic needs for energy and nutrition, dwelling primates preferentially feed on leaves, including mature ones, using a similar foraging strategy in response to habitat destruction, which consequently shapes the composition of their gut microbiota (Huang et al., 2008b; Wu et al., 2011). Firmicutes and Bacteroidetes likely make a large contribution to the digestion of plant polysaccharides, which are prominent in these primates’ leaf-based diets (Barelli et al., 2015; Su et al., 2016; Chen et al., 2020).

Specifically, the Firmicutes is linked to degrade dietary fiber (Sun et al., 2022), converting them to SCFAs that are directly absorbed by the host’s gut wall as energy (Turnbaugh et al., 2006). In addition, the Oscillospiraceae (Firmicutes) exhibits the highest relative abundance in both groups and could be involved in the degradation of mucin (Raimondi et al., 2021). Mucin is a major component of mucus in the gastrointestinal tract, which protects hosts from physical and chemical damage (Raimondi et al., 2021). Adequate mucin may assist in the smooth movement of hard dietary fiber in the gut, reducing the leakage risk of toxic secondary metabolites into the gut mucosa (Raimondi et al., 2021). However, abnormal elevation of mucin may be associated with inflammatory bowel disease (Raimondi et al., 2021). Oscillospiraceae, Christensenellaceae, and norank_o__Clostridia_UCG-014 are considered to be involved in the production of SCFAs (Morotomi et al., 2012; Koeck et al., 2014; Konikoff and Gophna, 2016). Additionally, Ruminococcaceae and Lachnospiraceae belonging to Firmicutes have often been reported in other studies and accounted for a relatively high proportion of our results too, making large contributions to the efficient degradation of plant polysaccharides (Arumugam et al., 2011; Tomova et al., 2019). Bacteroidetes is closely related to the degradation of protein and animal fat (Wu et al., 2011) and can degrade pectin and simple carbohydrates in fruits and other foods (Hale et al., 2019). Prevotellaceae (Bacteroidetes) is closely linked to the proportion of fruits in the animals’ food (Sun et al., 2016). These higher-abundance taxa are essential for the energy budget balance and for maintaining the health of white-headed black langurs in fragmented habitats.

The intersite variations of these two phyla in relative abundance can be expressed using F/B (ratio of the relative abundance of Firmicutes and Bacteroidetes; Turnbaugh et al., 2006). The folivorous primates, such as François’s langurs (8.24) (Chen et al., 2020) and Udzungwa red colobus monkeys (6.22) (Barelli et al., 2015), have higher F/B than frugivorous primates, such as red-fronted lemurs (*Eulemur rufifrons*) (0.98) (Murillo et al., 2022) and brown lemurs (*Eulemur fulvus*) (0.5) (Greene et al., 2021), as well as omnivorous primates, such as Tibetan macaques (*Macaca thibetana*) (2.65) (Xia et al., 2021). In this study, the F/B of white-headed black langurs was much higher than that of the aforementioned species (12.34). F/B is positively correlated with the ability to obtain energy (Turnbaugh et al., 2006). The higher F/B of the langurs may be associated with the fragmented habitat because they could have high energy requirements in response to the scarcity of high-quality foods caused by habitat fragmentation in the limestone forest.

In folivorous primates, although the major dominant gut microbiota are Firmicutes, Bacteroidetes, Actinobacteria, and Proteobacteria, there are minor differences in the order of their relative abundance. A tendency may be seen in the white-headed black langurs for the relative abundance of Proteobacteria or Actinobacteria to be higher than that of Bacteroidetes within a given situation (Que et al., 2022). Proteobacteria has been shown to facilitate the digestion of animal proteins (De Filippo et al., 2010). In parallel, this phylum includes many disease-causing genera; hence, its irregular and sharp elevations in the relative abundance frequently serve as the first reflection of an individual’s health status (Shin et al., 2015). Actinobacteria also play an essential role in maintaining gut homeostasis, of which, *Bifidobacteria*, the most widely recognized, is extensively used as a probiotic and can also assist in the hydrolysis of plant polysaccharides by producing glycosyl hydrolases (Pokusaeva et al., 2011). Gut microbiota increase the plasticity by adjusting their relative abundance to fit survival needs but not by changing the species of high-abundance taxa.

### 4.2. Intersite variations in gut microbiota

Our results indicate that the alpha diversity in the gut microbiota of the Bapen group was higher than that in the Banli group inhabiting more-fragmented sites, which is contrary to Prediction 1. The Banli group exhibits higher diet diversity because langurs in more-fragmented habitats adopt an energy conservation strategy and depend on more species of plants to obtain energy (Huang et al., 2017; Zhang et al., 2021). However, the Banli group did not display a high alpha diversity in the gut microbiota, as expected. This observation is contradictory to the findings of previous studies on black howler monkeys (Amato et al., 2013) and Udzungwa red colobus monkeys (Barelli et al., 2015), which considering gut microbiota diversity is positively corrected with diet diversity. This pattern could be associated with the degree of additional human disturbance suffered by the two groups and another indicator of diversity, namely, evenness. Additional human disturbance rather than habitat fragmentation itself may be responsible for the decrease in the diversity of gut microbiota (Fackelmann et al., 2021). The Banli area has more inhabitants and human activity, including farmland reclamation and felling of trees, encroaching on the home ranges of the langurs gradually (Huang et al., 2008a). This will further affect the langurs’ food resources by reducing the possibility that they eat parts of plants other than the leaves (Huang et al., 2008a). Although the Banli group fed on more kinds of plants, the common behavior of eating leaves may make the intake of nutrients unitary and thereby reduce the diversity of gut microbiota.

There is a significant difference in alpha diversity between the two groups; however, the richness did not markedly differ, suggesting that evenness may be contributed to this intersite variation in microbiota. The langurs’ response to severe fragmentation included the addition of lower-quality leaves, such as mature leaves, in their diets (Li et al., 2016). Meanwhile, the langurs are restricted by farmlands in the flat zones of limestone hills; hence, they occupy small home ranges and must increase their consumption of mature leaves (Huang et al., 2017). This behavior has led to the overwhelming dominance of Firmicutes and likely lowered the evenness of gut microbiota of the Banli group. Furthermore, higher vegetation diversity in the Bapen area increases the probability of foraging on fruits or other plant parts, resulting in an increased relative abundance of Bacteroidetes (Wu et al., 2011; Hale et al., 2019) and increased evenness.

The langurs in the Banli group had a higher abundance of Firmicutes and significantly differed from the Bapen group, which supports Prediction 2. This result may be related to the fact that young leaves are more likely to be exhausted in the more-fragmented Banli area, forcing the langurs to feed more on mature leaves (Li et al., 2016). Hence, the langurs of the Banli group might have a higher abundance of cellulose-degrading bacteria. Besides, the abundance of Firmicutes is also increased in high-fat, high-protein diet populations as it is closely associated with energy production (Turnbaugh et al., 2006). A greater F/B was found in the Banli group than in the Bapen group (18.07 vs. 5.75). The langurs of the Banli group experience more severer habitat fragmentation, which forces a reduction in home range size that requires them to increase movement time and daily path length to forage (Zhou et al., 2011; Zhang et al., 2021). Furthermore, the langurs in Banli spend more time on feeding compared with individuals in the Bapen group (Huang et al., 2017), which also requires the gut microbiota to efficiently degrade these fibers and convert them into energy.

Christensenellaceae forms a symbiotic system with other bacteria distributed in a probiotic that favors the maintenance of intestinal homeostasis (Li et al., 2020), and reduction of norank_o__Clostridia_UCG-014 has been observed in patients with diabetes (Karlsson et al., 2013). Both taxa are benefit to the host health and can breakdown cellulose to produce SCFAs (Morotomi et al., 2012; Koeck et al., 2014), which coincides with the adaptation of the Banli group to a more-fragmented habitat. Butyricicoccaceae contributes the most to intersite variations in the gut microbiota, which is a typical butyric acid-producing family and has significantly higher relative abundance in the microbiota from Banli group. This finding may be related to the differences in the parts of foods consumed by the two groups and the effect of strong interactions between colonies in the gut microbiota (Huang et al., 2017; Jeong and Kim, 2022). Studies have shown that the number of Butyricicoccaceae is negatively correlated with the proliferation of anaerobic methanogens (Jeong and Kim, 2022). The Bapen group eat less-mature leaves, which means that they need fewer methanogens to ferment cellulose in the digestive cavity (the structure of fermentable cellulose, similar to that of ruminants, evolved to accommodate diets rich in leaves; Lambert, 1998), which may lead to an increase in Butyricicoccaceae. Similarly, wild black howler monkeys have more Butyricicoccaceae in their gut microbiota, which is positively correlated with the consumption of young leaves by the hosts (Amato et al., 2015).

Notwithstanding the overwhelming advantage of Firmicutes, the contribution of Bacteroidetes to intersite variations could not be ignored. Most members of Bacteroidetes are likely affected by environmental factors because their change in abundance is only subject to a few explanations of host genetic factors (Goodrich et al., 2016). In this study, both Bacteroidetes and Prevotellaceae in the Bapen group were significantly higher than those in the Banli group. Functionally, Bacteroidetes actively participates in carbohydrate metabolism, which is characterized by the presence of the polysaccharide utilization locus, and its encoded proteins engage in the construction of carbohydrate utilization systems (Grondin et al., 2017). For example, during seasons rich in high-quality foods, the guts of rhesus macaques and Tibetan macaques are rich in Bacteroidetes associated with assimilating simple carbohydrates and pectin, of which Prevotellaceae is a representative (Li et al., 2021; Xia et al., 2021). The Bapen group suffered less habitat fragmentation and have larger home ranges (Huang et al., 2008a; Huang et al., 2017). This may allow them to eat higher-quality foods, such as fruits rich in simple carbohydrates, which further promote the colonization of Bacteroidetes.

The Banli group had more unique bacterial taxa. Even in poor habitats, the langurs are reluctant to settle for low-quality foods and search for a wider variety of plants with young leaves (Huang et al., 2017). High richness of unique bacterial taxa may increase the speed of recovery to the original state and the ability to maintain dynamic balance when the community is disturbed (Downing and Leibold, 2010; de Mazancourt et al., 2013). In addition, when langurs are deficient in important plant resources and increase the consumption of diversified supply plants that are not usually eaten, increased bacterial populations provided an excellent opportunity for functional redundancy. At this time, unique taxa with low abundance temporarily drive the digestion of these rare foods (Lozupone et al., 2012). This is of great significance for langurs with more interference in maintaining the stability of the gut microbiota because the degree of interference is often closely related to the susceptibility of the animals to diseases (Fackelmann et al., 2021).

The functional pathways that were significantly enriched at the KEGG pathway level 1 were detected in the Banli group, which may be related to the fact that they had a larger number of unique gut microbiota than the Banli group. This may address the need for the langurs in the Banli group to adjust to a more fragmented habitat by further expanding the feasibility of functional redundancy to take effect in the gut microbiota, as found in previous study on *Rana dybowskii* with diarrhoea (Tong et al., 2020). However, there were no significant difference in metabolic pathway. This may be related to the fact that white-headed black langurs maintain a highly foliar diet in limestone forest and the two study sites are highly fragmented (Huang et al., 2008a; Huang et al., 2017). Furthermore, among the metabolism-related pathways at level 2, two pathways linked to protein metabolism that were significantly enriched in the Bapen group. According to the composition of the gut microbiota in Bapen group of the langurs, this is probably because the Bacteroidetes and Proteobacteria closely related to protein degradation were significantly enriched. Further study should be needed.

### 4.3. Community assembly of the gut microbiota

Our results showed the greater influence of the stochastic process in the Banli group, which contradicts Prediction 3. The *R*^2^ of the NCM presented little difference between the two groups (both ~50%), which indicates that the community assembly of the gut microbiota of langurs is jointly built on a combination of stochastic and deterministic processes. Our results were similar to those of a study on latitudinal phytoplankton distribution (Chust et al., 2013), which suggests that deterministic or stochastic processes are not the exclusive outcome of community construction. The habitats of both groups have already become extremely fragmented, despite minor differences in fragmentation levels and result in similar environmental selection pressures on the langurs (Huang et al., 2008a). Furthermore, the flexible foraging behavior of langurs in the Banli group might narrow the gap with the physiological response of the langurs in the Bapen group, consequently bringing the processes affecting gut microbial community assembly in these two groups closer. The increased influence of the stochastic processes on the Banli group is grounded in the ascending environmental homogeneity and functional redundancy. Specifically, the small spatial fragments could decrease heterogeneity, reduce environmental preferences, and increase the possibility that stochastic factors will dominate (Bahram et al., 2016). Moreover, the strong functional redundancy caused by the high taxonomic richness increases the influence of stochastic factors (Vellend, 2010; Bahram et al., 2016). As specialists living in limestone forests, white-headed black langurs could have reached a peak in the richness of the gut microbiota for adaptation to poor habitats.

The migration rates of the Bapen group were only slightly higher than those of the Banli group. Species migration rates are generally positively correlated with their diversity as they contribute heavily to quantitative dispersal (Mo et al., 2018). More frequent group activities of hosts may shrink the social distance between individuals, which is conducive to the spread of gut microbiota among langurs (Sarkar et al., 2020). Intimate communicative behaviors between individual langurs living in the limestone forest include playing and grooming, with the former occurring more frequently among young langurs and the latter being observed more continually on colder days (Huang et al., 2017; Zheng et al., 2021). The time budgets of these two groups of langurs in grooming are similar, but individuals in the Banli group spend more time playing with each other (Huang et al., 2017). Therefore, compared with the Banli group, the langurs in the Bapen group showed only a weak advantage in the interindividual migration rate of the gut microbiota. More than 50% of the bacterial taxa in the Banli and Bapen groups fell within the 95% confidence interval, which indicates that they were mostly in a stochastic condition. It is still necessary to consider the degree of fragmentation in both sites. Severe fragmentation has resulted in progressively smaller habitat fragments for both groups, which is likely to enhance environmental homogeneity. These similar indices in the NCM not only suggest that deterministic and stochastic processes are of equal importance but also indicate that the habitats are severely fragmented and that population and habitat conservation activities are urgently needed.

There should be weakness in current study. Specifically, we focused on the intersite variation in the microbiota, without considering their seasonality. We admit that current results could be weakened by the detailed seasonal comparison; however, we provide preliminary pattern of microbiota composition and structure for the karst dwelling primates, consequently deepening our understanding on the adaptation in responses to habitat fragmentation on the physiological insight. Moreover, information on the function of the microbiota is relatively limited. Further studies at the metagenomic level and the interaction between seasonality and geography should be needed.

In summary, intersite variations exist in the structure and diversity of gut microbiota between geographical groups of white-headed black langurs inhabiting various forests under different levels of habitat fragmentation. The gut microbiota diversity of langurs in Banli group with deeper fragmentation was lower, which could be related to increased human disturbance. The Banli group had much higher levels of Firmicutes, which could be related to their consumption of low-quality foods, such as mature leaves. Firmicutes helps the langurs digest dietary fiber and produce large amounts of energy, which helps them adapt to life in the limestone forest. Both groups showed similar values of interpretive degree and were at ~50% on the community assembly of gut microbiota. This finding indicates that the community assembly is built on a combination of stochastic and deterministic processes, which could be related to the flexible survival strategy of the Banli group narrowing the gap with the Bapen group. We conclude that the white-headed black langurs’ response to changing food resources in habitats with different levels of fragmentation is to adjust the diversity and composition of the gut microbiota. This finding highlights the importance of gut microbiota in the adaptation to habitats and the need for using physiological indicators to study the mechanisms by which wildlife responds to human disturbance and/or ecological variability.

## Data availability statement

The datasets presented in this study can be found in online repositories. The names of the repository/repositories and accession number(s) can be found at: https://www.ncbi.nlm.nih.gov/, PRJNA904436.

## Ethics statement

The animal study was reviewed and approved by Administration Center of Guangxi Chongzuo White-headed Langur National Nature Reserve.

## Author contributions

ZH designed the study. YiL and YC analyzed the data. YiL wrote the manuscript. JZ, ZL, DN, and JL collected samples. YoL and ZH revised the manuscript. All authors read and approved the submitted manuscript.

## Funding

This work was supported by the National Natural Science Foundation of China (32170488, 31960106, and 31960104).

## Conflict of interest

The authors declare that the research was conducted in the absence of any commercial or financial relationships that could be construed as a potential conflict of interest.

## Publisher’s note

All claims expressed in this article are solely those of the authors and do not necessarily represent those of their affiliated organizations, or those of the publisher, the editors and the reviewers. Any product that may be evaluated in this article, or claim that may be made by its manufacturer, is not guaranteed or endorsed by the publisher.

## Footnotes

^1^ https://www.majorbio.com/

^2^ https://ccb.jhu.edu/software/FLASH/index.shtml

^3^ http://www.drive5.com/usearch/

^4^ http://www.drive5.com/uparse/

^5^ http://qiime.org/install/index.html

^6^ https://www.arb-silva.de/

^7^ http://rdp.cme.msu.edu/

^8^ https://www.mothur.org/wiki/Download_mothur

^9^ https://github.com/picrust/picrust2/

## Supplementary material

Supplementary Figure 1 Shannon **(A)** and Sob **(B)** dilution curves show sufficient sequencing depth.

Supplementary Figure 2 Number of species shared and unique of Banli and Bapen groups at OTU **(A)**, phylum **(B)**, and family **(C)** levels.

## References

[ref1] Amato K. R. Leigh S. R. Kent A. Mackie R. I. Yeoman C. J. Stumpf R. M.. (2015). The gut microbiota appears to compensate for seasonal diet variation in the wild black howler monkey (*Alouatta pigra*). Microb. Ecol. 69, 434–443. doi: 10.1007/s00248-014-0554-7, PMID: 25524570

[ref2] Amato K. R. Yeoman C. J. Kent A. Righini N. Carbonero F. Estrada A.. (2013). Habitat degradation impacts black howler monkey (*Alouatta pigra*) gastrointestinal microbiomes. ISME J. 7, 1344–1353. doi: 10.1038/ismej.2013.16, PMID: 23486247 PMC3695285

[ref3] Arumugam M. Raes J. Pelletier E. Le Paslier D. Yamada T. Mende D. R.. (2011). Enterotypes of the human gut microbiome. Nature 473, 174–180. doi: 10.1038/nature09944, PMID: 21508958 PMC3728647

[ref4] Bahram M. Kohout P. Anslan S. Harend H. Abarenkov K. Tedersoo L. (2016). Stochastic distribution of small soil eukaryotes resulting from high dispersal and drift in a local environment. ISME J. 10, 885–896. doi: 10.1038/ismej.2015.164, PMID: 26394006 PMC4796928

[ref5] Barelli C. Albanese D. Donati C. Pindo M. Dallago C. Rovero F.. (2015). Habitat fragmentation is associated to gut microbiota diversity of an endangered primate: implications for conservation. Sci. Rep. 5:14862. doi: 10.1038/srep14862, PMID: 26445280 PMC4595646

[ref6] Bleisch B. Long Y. (2020). Trachypithecus leucocephalus. The IUCN Red List of Threatened Species 2020. doi: 10.2305/IUCN.UK.2020-2.RLTS.T39872A17988378.en (Accessed October 10, 2022).

[ref7] Breiman L. (2001). Random forests. Mach. Learn. 45, 5–32. doi: 10.1023/A:1010933404324

[ref8] Caporaso J. G. Kuczynski J. Stombaugh J. Bittinger K. Bushman F. D. Costello E. K.. (2010). QIIME allows analysis of high-throughput community sequencing data. Nat. Methods 7, 335–336. doi: 10.1038/nmeth.f.303, PMID: 20383131 PMC3156573

[ref9] Chen T. Li Y. H. Liang J. P. Li Y. B. Huang Z. H. (2020). Variations in the gut microbiota of sympatric François’ langurs and rhesus macaques living in limestone forests in Southwest Guangxi. China. Glob. Ecol. Conserv. 22:e00929. doi: 10.1016/j.gecco.2020.e00929

[ref10] Chen W. D. Ren K. X. Isabwe A. Chen H. H. Liu M. Yang J. (2019). Stochastic processes shape microeukaryotic community assembly in a subtropical river across wet and dry seasons. Microbiome 7:138. doi: 10.1186/s40168-019-0763-x, PMID: 31640783 PMC6806580

[ref11] Chust G. Irigoien X. Chave J. Harris R. P. (2013). Latitudinal phytoplankton distribution and the neutral theory of biodiversity. Glob. Ecol. Biogeogr. 22, 531–543. doi: 10.1111/geb.12016

[ref12] Cryan J. F. O’Riordan K. J. Cowan C. S. M. Sandhu K. V. Bastiaanssen T. F. S. Boehme M.. (2019). The microbiota-gut-brain axis. Physiol. Rev. 99, 1877–2013. doi: 10.1152/physrev.00018.2018 31460832

[ref13] De Filippis F. Pellegrini N. Vannini L. Jeffery I. B. La Storia A. Laghi L.. (2016). High-level adherence to a Mediterranean diet beneficially impacts the gut microbiota and associated metabolome. Gut 65, 1812–1821. doi: 10.1136/gutjnl-2015-309957, PMID: 26416813

[ref14] De Filippo C. Cavalieri D. Di Paola M. Ramazzotti M. Poullet J. B. Massart S.. (2010). Impact of diet in shaping gut microbiota revealed by a comparative study in children from Europe and rural Africa. Proc. Natl. Acad. Sci. U. S. A. 107, 14691–14696. doi: 10.1073/pnas.1005963107, PMID: 20679230 PMC2930426

[ref15] de Mazancourt C. Isbell F. Larocque A. Berendse F. De Luca E. Grace J. B.. (2013). Predicting ecosystem stability from community composition and biodiversity. Ecol. Lett. 16, 617–625. doi: 10.1111/ele.12088 23438189

[ref16] Deehan E. C. Yang C. Perez-Muñoz M. E. Nguyen N. K. Cheng C. C. Triador L.. (2020). Precision microbiome modulation with discrete dietary fiber structures directs short-chain fatty acid production. Cell Host Microbe 27, 389–404.e6. doi: 10.1016/j.chom.2020.01.006, PMID: 32004499

[ref17] Dini-Andreote F. Stegen J. C. Van Elsas J. D. Salles J. F. (2015). Disentangling mechanisms that mediate the balance between stochastic and deterministic processes in microbial succession. Proc. Natl. Acad. Sci. U. S. A. 112, E1326–E1332. doi: 10.1073/pnas.1414261112, PMID: 25733885 PMC4371938

[ref18] Dominguez-Bello M. G. Costello E. K. Contreras M. Magris M. Hidalgo G. Fierer N.. (2010). Delivery mode shapes the acquisition and structure of the initial microbiota across multiple body habitats in newborns. Proc. Natl. Acad. Sci. U. S. A. 107, 11971–11975. doi: 10.1073/pnas.1002601107, PMID: 20566857 PMC2900693

[ref001] Douglas G. M. Maffei V. J. Zaneveld J. R. Yurgel S. N. Brown J. R. Taylor C. M.. (2020). PICRUSt2 for prediction of metagenome functions. Nat. Biotechnol. 38, 685–688. doi: 10.1038/S41587-020-0548-6 32483366 PMC7365738

[ref19] Downing A. L. Leibold M. A. (2010). Species richness facilitates ecosystem resilience in aquatic food webs. Freshwater Bio. 55, 2123–2137. doi: 10.1111/j.1365-2427.2010.02472.x

[ref20] Edgar R. C. (2010). Search and clustering orders of magnitude faster than BLAST. Bioinformatics 26, 2460–2461. doi: 10.1093/bioinformatics/btq461, PMID: 20709691

[ref21] Edgar R. C. (2013). UPARSE: highly accurate OTU sequences from microbial amplicon reads. Nat. Methods 10, 996–998. doi: 10.1038/NMETH.2604, PMID: 23955772

[ref22] Edgar R. C. Haas B. J. Clemente J. C. Quince C. Knight R. (2011). UCHIME improves sensitivity and speed of chimera detection. Bioinformatics 27, 2194–2200. doi: 10.1093/bioinformatics/btr381, PMID: 21700674 PMC3150044

[ref23] Fackelmann G. Gillingham M. A. F. Schmid J. Heni A. C. Wilhelm K. Schwensow N.. (2021). Human encroachment into wildlife gut microbiomes. Commun. Biol. 4:800. doi: 10.1038/s42003-021-02315-7, PMID: 34172822 PMC8233340

[ref24] Fargione J. Brown C. S. Tilman D. (2003). Community assembly and invasion: an experimental test of neutral versus niche processes. Proc. Natl. Acad. Sci. U. S. A. 100, 8916–8920. doi: 10.1073/pnas.1033107100, PMID: 12843401 PMC166413

[ref25] Frankel J. S. Mallott E. K. Hopper L. M. Ross S. R. Amato K. R. (2019). The effect of captivity on the primate gut microbiome varies with host dietary niche. Am. J. Primatol. 81:e23061. doi: 10.1002/ajp.23061, PMID: 31713260

[ref26] Gad M. Hou L. Y. Li J. W. Wu Y. Rashid A. Chen N. W.. (2020). Distinct mechanisms underlying the assembly of microeukaryotic generalists and specialists in an anthropogenically impacted river. Sci. Total Environ. 748:141434. doi: 10.1016/j.scitotenv.2020.141434, PMID: 32814298

[ref27] Goodrich J. K. Davenport E. R. Beaumont M. Jackson M. A. Knight R. Ober C.. (2016). Genetic determinants of the gut microbiome in UK twins. Cell Host Microbe 19, 731–743. doi: 10.1016/j.chom.2016.04.017, PMID: 27173935 PMC4915943

[ref28] Greene L. K. Rambeloson E. Rasoanaivo H. A. Foss E. D. Yoder A. D. Drea C. M.. (2021). Gut microbial diversity and ecological specialization in four sympatric lemur species under lean conditions. Int. J. Primatol. 42, 961–979. doi: 10.1007/s10764-021-00257-9

[ref29] Groer M. W. Luciano A. A. Dishaw L. J. Ashmeade T. L. Miller E. Gilbert J. A. (2014). Development of the preterm infant gut microbiome: a research priority. Microbiome 2:38. doi: 10.1186/2049-2618-2-38, PMID: 25332768 PMC4203464

[ref30] Grondin J. M. Tamura K. Déjean G. Abbott D. W. Brumer H. (2017). Polysaccharide utilization loci: fueling microbial communities. J. Bacteriol. 199:e00860. doi: 10.1128/JB.00860-16, PMID: 28138099 PMC5512228

[ref31] Guangxi Forestry Department (1993). Nature reserves in Guangxi. Beijing: China Forestry Publishing House.

[ref32] Hale V. L. Tan C. L. Niu K. F. Yang Y. Q. Zhang Q. K. Knight R.. (2019). Gut microbiota in wild and captive Guizhou snub-nosed monkeys, *Rhinopithecus brelichi*. Am. J. Primatol. 81:e22989. doi: 10.1002/ajp.22989, PMID: 31106872

[ref33] Heiman M. L. Greenway F. L. (2016). A healthy gastrointestinal microbiome is dependent on dietary diversity. Mol. Metab. 5, 317–320. doi: 10.1016/j.molmet.2016.02.005, PMID: 27110483 PMC4837298

[ref34] Heys C. Cheaib B. Busetti A. Kazlauskaite R. Maier L. Sloan W. T.. (2020). Neutral processes dominate microbial community assembly in Atlantic Salmon, *Salmo salar*. Appl. Environ. Microbiol. 86:e02283–19. doi: 10.1128/AEM.02283-19, PMID: 32033945 PMC7117918

[ref35] Huang C. M. (2002). White-headed langur in China. Guilin: Guangxi Normal University Press.

[ref36] Huang C. M. Li Y. B. Zhou Q. H. Feng Y. X. Chen Z. Yu H.. (2008a). Karst habitat fragmentation and the conservation of the white-headed langur (*Trachypithecus leucocephalus*) in China. Primate Conserv. 23, 133–139. doi: 10.1896/052.023.0116

[ref37] Huang C. M. Wu H. Zhou Q. H. Li Y. B. Cai X. W. (2008b). Feeding strategy of François’ langur and white-headed langur at Fusui, China. Am. J. Primatol. 70, 320–326. doi: 10.1002/ajp.20490, PMID: 17924424

[ref38] Huang Z. H. Yuan P. S. Huang H. L. Tang X. P. Xu W. J. Huang C. M.. (2017). Effect of habitat fragmentation on ranging behavior of white-headed langurs in limestone forests in Southwest China. Primates 58, 423–434. doi: 10.1007/s10329-017-0600-4, PMID: 28197795

[ref39] Jeong S. Y. Kim T. G. (2022). Determination of methanogenesis by nutrient availability via regulating the relative fitness of methanogens in anaerobic digestion. Sci. Total Environ. 838:156002. doi: 10.1016/j.scitotenv.2022.156002, PMID: 35588829

[ref40] Karlsson F. H. Tremaroli V. Nookaew I. Bergström G. Behre C. J. Fagerberg B.. (2013). Gut metagenome in European women with normal, impaired and diabetic glucose control. Nature 498, 99–103. doi: 10.1038/nature12198, PMID: 23719380

[ref41] Koeck D. E. Pechtl A. Zverlov V. V. Schwarz W. H. (2014). Genomics of cellulolytic bacteria. Curr. Opin. Biotechnol. 29, 171–183. doi: 10.1016/j.copbio.2014.07.002 25104562

[ref42] Konikoff T. Gophna U. (2016). *Oscillospira*: a central, enigmatic component of the human gut microbiota. Trends Microbiol. 24, 523–524. doi: 10.1016/j.tim.2016.02.015, PMID: 26996766

[ref43] Lambert J. E. (1998). Primate digestion: interactions among anatomy, physiology, and feeding ecology. Evol. Anthropol. Issues News Rev. 7, 8–20. doi: 10.1002/(SICI)1520-6505(1998)7:1<8::AID-EVAN3>3.0.CO;2-C

[ref44] Le H. T. Hoang D. M. Covert H. H. (2019). Diet of the Indochinese silvered langur (*Trachypithecus germaini*) in Kien Luong karst area, Kien Giang Province. Am. J. Primatol. 81:e23041. doi: 10.1002/ajp.23041, PMID: 31436340

[ref45] Ley R. E. Hamady M. Lozupone C. Turnbaugh P. J. Ramey R. R. Bircher J. S.. (2008). Evolution of mammals and their gut microbes. Science 320, 1647–1651. doi: 10.1126/science.1155725, PMID: 18497261 PMC2649005

[ref46] Li Y. H. Chen T. Liang J. P. Li Y. B. Huang Z. H. (2021). Seasonal variation in the gut microbiota of rhesus macaques inhabiting limestone forests of Southwest Guangxi, China. Arch. Microbiol. 203, 787–798. doi: 10.1007/s00203-020-02069-6, PMID: 33057745

[ref47] Li B. Gao H. M. Song P. S. Liang C. B. Jiang F. Xu B.. (2022). Captivity shifts gut microbiota communities in white-lipped deer (*Cervus albirostris*). Animals 12:431. doi: 10.3390/ani12040431, PMID: 35203139 PMC8868073

[ref48] Li X. Li Z. W. He Y. Li P. Zhou H. W. Zeng N. Y. (2020). Regional distribution of Christensenellaceae and its associations with metabolic syndrome based on a population-level analysis. PeerJ 8:e9591. doi: 10.7717/peerj.9591, PMID: 32832265 PMC7413085

[ref49] Li Z. Y. Rogers M. E. (2005). Habitat quality and range use of white-headed langurs in Fusui, China. Folia primatol. 76, 185–195. doi: 10.1159/000086020, PMID: 16088186

[ref50] Li D. Y. Yuan P. S. Krzton A. Huang C. M. Zhou Q. H. (2016). Dietary adaptation of white-headed langurs in a fragmented limestone habitat. Mammalia 80, 153–162. doi: 10.1515/mammalia-2014-0152

[ref51] Li H. Zhou R. Zhu J. X. Huang X. D. Qu J. P. (2019). Environmental filtering increases with elevation for the assembly of gut microbiota in wild pikas. Microb. Biotechnol. 12, 976–992. doi: 10.1111/1751-7915.13450, PMID: 31380612 PMC6680628

[ref52] Liu H. Y. Chen T. Li Y. H. Zheng J. J. Liu Z. Li Y. B.. (2022). Seasonal variations in gut microbiota of semiprovisioned rhesus macaques (*Macaca mulatta*) living in a limestone forest of Guangxi, China. Front. Microbiol. 13:951507. doi: 10.3389/fmicb.2022.951507, PMID: 36204603 PMC9530203

[ref53] Liu J. J. Cui X. Liu Z. X. Guo Z. K. Yu Z. H. Yao Q.. (2019). The diversity and geographic distribution of cultivable bacillus-like bacteria across black soils of Northeast China. Front. Microbiol. 10:1424. doi: 10.3389/fmicb.2019.01424, PMID: 31293554 PMC6598460

[ref54] Lozupone C. A. Stombaugh J. I. Gordon J. I. Jansson J. K. Knight R. (2012). Diversity, stability and resilience of the human gut microbiota. Nature 489, 220–230. doi: 10.1038/nature11550, PMID: 22972295 PMC3577372

[ref55] Magoč T. Salzberg S. L. (2011). FLASH: fast length adjustment of short reads to improve genome assemblies. Bioinformatics 27, 2957–2963. doi: 10.1093/bioinformatics/btr507, PMID: 21903629 PMC3198573

[ref56] Matson V. Chervin C. S. Gajewski T. F. (2021). Cancer and the microbiome-influence of the commensal microbiota on cancer, immune responses, and immunotherapy. Gastroenterology 160, 600–613. doi: 10.1053/j.gastro.2020.11.041, PMID: 33253684 PMC8409239

[ref57] McManus N. Holmes S. M. Louis E. E. Johnson S. E. Baden A. L. Amato K. R. (2021). The gut microbiome as an indicator of habitat disturbance in a critically endangered lemur. BMC Ecol. Evol. 21:222. doi: 10.1186/s12862-021-01945-z, PMID: 34915861 PMC8680155

[ref58] Mo Y. Y. Zhang W. J. Yang J. Lin Y. S. Yu Z. Lin S. J. (2018). Biogeographic patterns of abundant and rare bacterioplankton in three subtropical bays resulting from selective and neutral processes. ISME J. 12, 2198–2210. doi: 10.1038/s41396-018-0153-6, PMID: 29880912 PMC6092436

[ref59] Mori H. Maruyama F. Kato H. Toyoda A. Dozono A. Ohtsubo Y.. (2014). Design and experimental application of a novel non-degenerate universal primer set that amplifies prokaryotic 16S rRNA genes with a low possibility to amplify eukaryotic rRNA genes. DNA Res. 21, 217–227. doi: 10.1093/dnares/dst052, PMID: 24277737 PMC3989492

[ref60] Morotomi M. Nagai F. Watanabe Y. (2012). Description of *Christensenella minuta* gen. Nov., sp. nov., isolated from human faeces, which forms a distinct branch in the order Clostridiales, and proposal of Christensenellaceae fam. Nov. Evol. Microbiol. 62, 144–149. doi: 10.1099/ijs.0.026989-0, PMID: 21357455

[ref61] Murillo T. Schneider D. Fichtel C. Daniel R. (2022). Dietary shifts and social interactions drive temporal fluctuations of the gut microbiome from wild redfronted lemurs. ISME Commun. 2:3. doi: 10.1038/s43705-021-00086-0 PMC9723586 37938637

[ref62] Pokusaeva K. Fitzgerald G. F. van Sinderen D. (2011). Carbohydrate metabolism in *Bifidobacteria*. Genes Nutr. 6, 285–306. doi: 10.1007/s12263-010-0206-6, PMID: 21484167 PMC3145055

[ref63] Que T. C. Pang X. W. Huang H. L. Chen P. Y. Wei Y. F. Hua Y. M.. (2022). Comparative gut microbiome in *Trachypithecus leucocephalus* and other primates in Guangxi, China, based on metagenome sequencing. Front. Cell. Infect. Microbiol. 12:872841. doi: 10.3389/fcimb.2022.872841, PMID: 35601103 PMC9114771

[ref64] Raimondi S. Musmeci E. Candeliere F. Amaretti A. Rossi M. (2021). Identification of mucin degraders of the human gut microbiota. Sci. Rep. 11:11094. doi: 10.1038/s41598-021-90553-4, PMID: 34045537 PMC8159939

[ref65] Robin X. Turck N. Hainard A. Tiberti N. Lisacek F. Sanchez J. C.. (2011). pROC: an open-source package for R and S+ to analyze and compare ROC curves. BMC Bioinformatics 12:77. doi: 10.1186/1471-2105-12-77, PMID: 21414208 PMC3068975

[ref66] Rothschild D. Weissbrod O. Barkan E. Kurilshikov A. Korem T. Zeevi D.. (2018). Environment dominates over host genetics in shaping human gut microbiota. Nature 555, 210–215. doi: 10.1038/nature25973, PMID: 29489753

[ref67] Sarkar A. Harty S. Johnson K. V. Moeller A. H. Archie E. A. Schell L. D.. (2020). Microbial transmission in animal social networks and the social microbiome. Nat. Ecol. Evol. 4, 1020–1035. doi: 10.1038/s41559-020-1220-8, PMID: 32572221

[ref68] Shin N. R. Whon T. W. Bae J. W. (2015). Proteobacteria: microbial signature of dysbiosis in gut microbiota. Trends Biotechnol. 33, 496–503. doi: 10.1016/j.tibtech.2015.06.011 26210164

[ref69] Sloan W. T. Lunn M. Woodcock S. Head I. M. Nee S. Curtis T. P. (2006). Quantifying the roles of immigration and chance in shaping prokaryote community structure. Environ. Microbiol. 8, 732–740. doi: 10.1111/j.1462-2920.2005.00956.x, PMID: 16584484

[ref70] Su C. Zuo R. J. Liu W. W. Sun Y. Li Z. Jin X. L.. (2016). Fecal bacterial composition of Sichuan snub-nosed monkeys (*Rhinopithecus roxellana*). Int. J. Primatol. 37, 518–533. doi: 10.1007/s10764-016-9918-9

[ref71] Sun B. Wang X. Bernstein S. Huffman M. A. Xia D. P. Gu Z. Y.. (2016). Marked variation between winter and spring gut microbiota in free-ranging Tibetan macaques (*Macaca thibetana*). Sci. Rep. 6:26035. doi: 10.1038/srep26035, PMID: 27180722 PMC4867428

[ref72] Sun Y. G. Zhang S. S. Nie Q. X. He H. J. Tan H. Z. Geng F.. (2022). Gut firmicutes: relationship with dietary fiber and role in host homeostasis. Crit. Rev. Food Sci. Nutr. 1–16. doi: 10.1080/10408398.2022.2098249, PMID: 35822206

[ref73] Szekely B. A. Singh J. Marsh T. L. Hagedorn C. Werre S. R. Kaur T. (2010). Fecal bacterial diversity of human-habituated wild chimpanzees (*Pan troglodytes schweinfurthii*) at Mahale Mountains National Park, Western Tanzania. Am. J. Primatol. 72, 566–574. doi: 10.1002/ajp.20809, PMID: 20146237

[ref74] Tomova A. Bukovsky I. Rembert E. Yonas W. Alwarith J. Barnard N. D.. (2019). The effects of vegetarian and vegan diets on gut microbiota. Front. Nutr. 6:47. doi: 10.3389/fnut.2019.00047, PMID: 31058160 PMC6478664

[ref75] Tong Q. Cui L. Y. Du X. P. Hu Z. F. Bie J. Xiao J. H.. (2020). Comparison of gut microbiota diversity and predicted functions between healthy and diseased captive *Rana dybowskii*. Front. Microbiol. 11:2096. doi: 10.3389/fmicb.2020.02096, PMID: 32983063 PMC7490342

[ref76] Trosvik P. Rueness E. K. de Muinck E. J. Moges A. Mekonnen A. (2018). Ecological plasticity in the gastrointestinal microbiomes of Ethiopian *Chlorocebus* monkeys. Sci. Rep. 8:20. doi: 10.1038/s41598-017-18435-2, PMID: 29311667 PMC5758796

[ref77] Turnbaugh P. J. Ley R. E. Mahowald M. A. Magrini V. Mardis E. R. Gordon J. I. (2006). An obesity-associated gut microbiome with increased capacity for energy harvest. Nature 444, 1027–1031. doi: 10.1038/nature05414, PMID: 17183312

[ref78] Vellend M. (2010). Conceptual synthesis in community ecology. Q. Rev. Biol. 85, 183–206. doi: 10.1086/652373 20565040

[ref79] Warton D. I. Hui F. K. (2010). The arcsine is asinine: the analysis of proportions in ecology. Ecology 92, 3–10. doi: 10.1890/10-0340.1, PMID: 21560670

[ref80] Wasimuddin M. H. Ratovonamana Y. R. Rakotondranary S. J. Ganzhorn J. U. Sommer S. (2022). Anthropogenic disturbance impacts gut microbiome homeostasis in a Malagasy primate. Front. Microbiol. 13:911275. doi: 10.3389/fmicb.2022.911275, PMID: 35801106 PMC9253676

[ref81] Wong B. M. Candolin U. (2015). Behavioral responses to changing environments. Behav. Ecol. 26, 665–673. doi: 10.1093/beheco/aru183

[ref82] Wu G. D. Chen J. Hoffmann C. Bittinger K. Chen Y. Y. Keilbaugh S. A.. (2011). Linking long-term dietary patterns with gut microbial enterotypes. Science 334, 105–108. doi: 10.1126/science.1208344, PMID: 21885731 PMC3368382

[ref83] Xia T. R. Yao Y. F. Wang C. Dong M. M. Wu Y. H. Li D. Y.. (2021). Seasonal dynamics of gut microbiota in a cohort of wild Tibetan macaques (*Macaca thibetana*) in western China. Glob. Ecol. Conserv. 25:e01409. doi: 10.1016/j.gecco.2020.e01409

[ref84] Zhang K. C. Zhou Q. H. Xu H. L. Huang Z. H. (2021). Diet, food availability, and climatic factors drive ranging behavior in white-headed langurs in the limestone forests of Guangxi, Southwest China. Zool. Res. 42, 406–411. doi: 10.24272/j.issn.2095-8137.2020.292, PMID: 34075733 PMC8317190

[ref85] Zhao J. S. Yao Y. F. Li D. Y. Xu H. M. Wu J. Y. Wen A. X.. (2018). Characterization of the gut microbiota in six geographical populations of Chinese rhesus macaques (*Macaca mulatta*), implying an adaptation to high-altitude environment. Microb. Ecol. 76, 565–577. doi: 10.1007/s00248-018-1146-8, PMID: 29372281

[ref86] Zheng J. J. Zhang K. C. Liang J. P. Li Y. B. Huang Z. H. (2021). Food availability, temperature, and day length drive seasonal variations in the positional behavior of white-headed langurs in the limestone forests of Southwest Guangxi. China. Ecol. Evol. 11, 14857–14872. doi: 10.1002/ece3.8171, PMID: 34765146 PMC8571639

[ref87] Zhou J. Z. Liu W. Z. Deng Y. Jiang Y. H. Xue K. He Z. L.. (2013). Stochastic assembly leads to alternative communities with distinct functions in a bioreactor microbial community. mBio 4:e00584-12. doi: 10.1128/mbio.00584-12, PMID: 23462114 PMC3585448

[ref88] Zhou J. Z. Ning D. L. (2017). Stochastic community assembly: does it matter in microbial ecology? Microbiol. Mol. Biol. R. 81:e00002–17. doi: 10.1128/MMBR.00002-17, PMID: 29021219 PMC5706748

[ref89] Zhou Q. H. Tang X. P. Huang H. L. Huang C. M. (2011). Factors affecting the ranging behavior of white-headed langurs (*Trachypithecus leucocephalus*). Int. J. Primatol. 32, 511–523. doi: 10.1007/s10764-010-9486-3

