# Supplementary figures and images for: Gut microbiota of white-headed black langurs (Trachypithecus leucocephalus) in responses to habitat fragmentation
Source: Front Microbiol. 2023 Feb 13;14:1126257. doi: 10.3389/fmicb.2023.1126257 (PMC9968942; doi:10.3389/fmicb.2023.1126257)

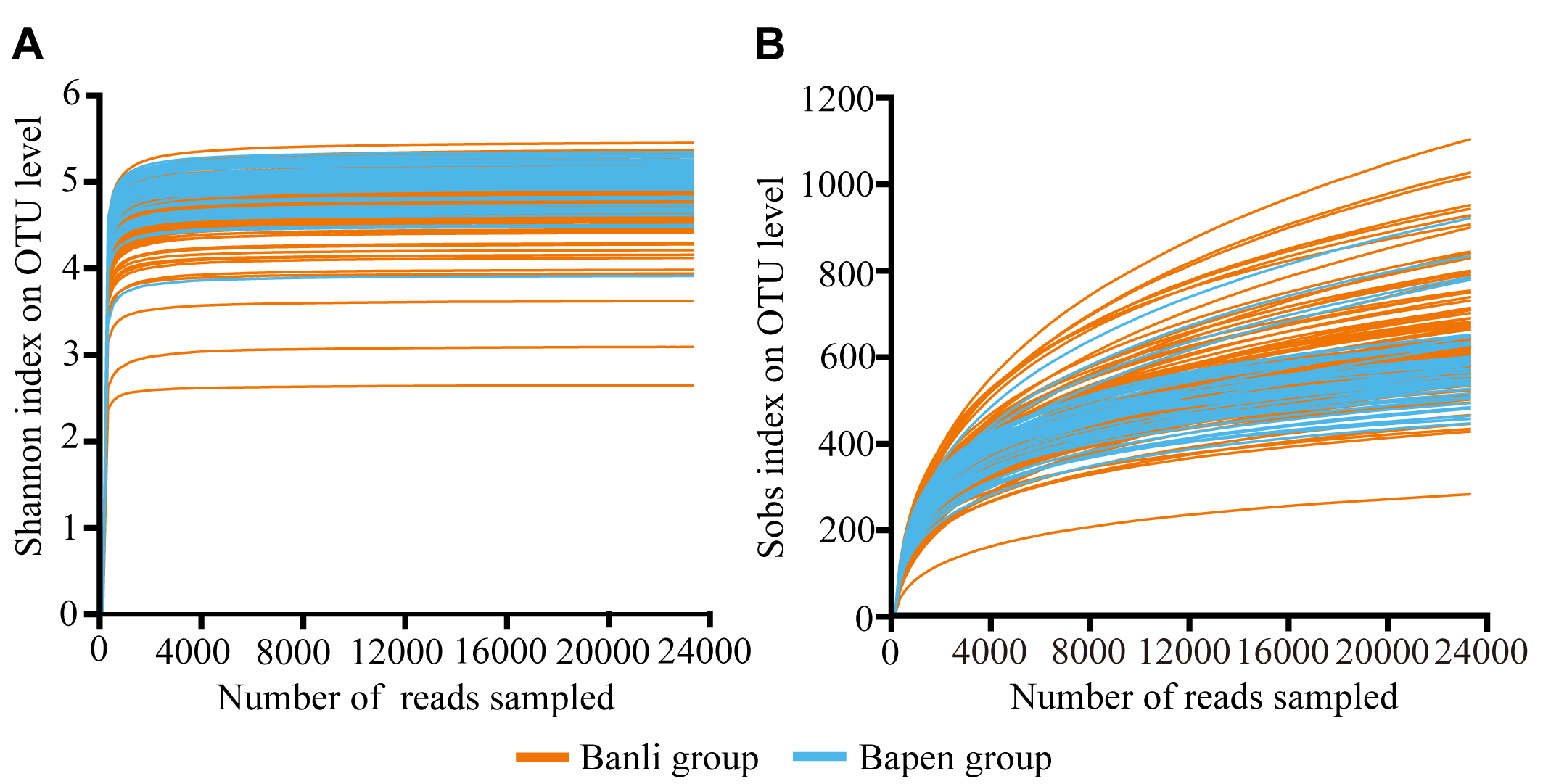

Supplement: Supplementary Figure 1 — Shannon (A) and Sob (B) dilution curves show sufficient sequencing depth. [file Image_1.TIF]

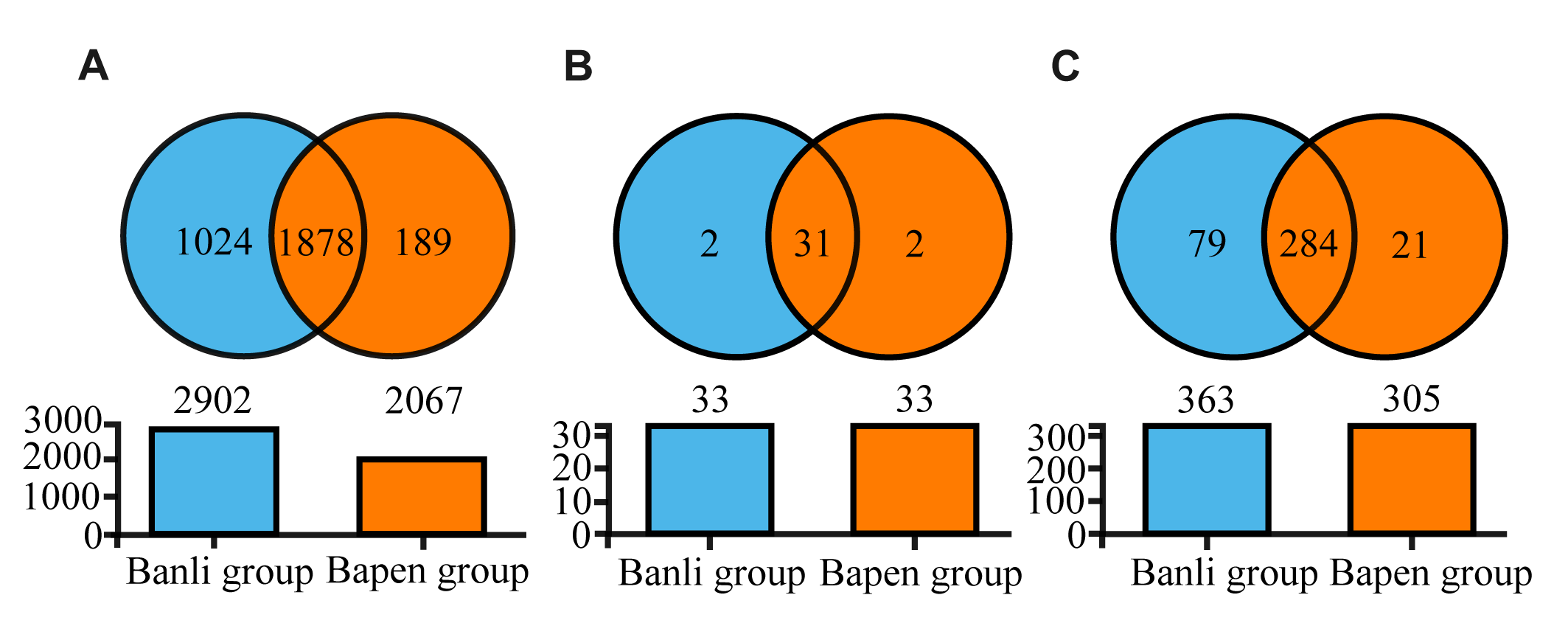

Supplement: Supplementary Figure 2 — Number of species shared and unique of Banli and Bapen groups at OTU (A), phylum (B), and family (C) levels. [file Image_2.TIF]
