# Supplementary material for: Gut microbiota of white-headed black langurs (Trachypithecus leucocephalus) in responses to habitat fragmentation
Source: Front Microbiol. 2023 Feb 13;14:1126257. doi: 10.3389/fmicb.2023.1126257 (PMC9968942; doi:10.3389/fmicb.2023.1126257)
Supplement: Supplementary file 3 [file Table_1.DOCX]

**Information on fecal samples of white-headed black langurs**

| Sampling site | Sampling time | Group | Number of samples | Sample number |
| --- | --- | --- | --- | --- |
| Banli area | 2020.12 | BG group | 10 | BL021-BL030 |
|  |  | NS group | 10 | BL031-BL040 |
|  |  | SKS group | 11 | BL053-BL063 |
|  | 2021.01 | BG group | 12 | BL064-BL075 |
|  |  | NS group | 12 | BL089-BL100 |
|  |  | SKS group | 12 | BL113-BL124 |
|  | 2021.06 | BG group | 15 | BL276-BL290 |
|  |  | NS group | 14 | BL291-BL304 |
|  |  | SKS group | 14 | BL305-BL318 |
|  | 2021.07 | BG group | 16 | BL331-BL346 |
|  |  | NS group | 14 | BL359-BL372 |
|  |  | SKS group | 15 | BL373-BL387 |
| Bapen area | 2021.07 | NCB group | 7 | BP001-BP007 |
|  |  | GH group | 7 | BP008-BP014 |
|  |  | NCYL group | 6 | BP015-BP020 |
|  | 2021.11 | NCW group | 8 | BP021-BP028 |
|  |  | BNTZ group | 5 | BP029-BP033 |
|  |  | NN3 group | 8 | BP034-BP041 |
|  |  | GH group | 7 | BP042-BP048 |
